# Left and right ventricular kinetic energy using time-resolved versus time-average ventricular volumes

**DOI:** 10.1186/1532-429X-17-S1-P67

**Published:** 2015-02-03

**Authors:** Syed F Hussaini, Alejandro Roldán-Alzate, Christopher J Francois

**Affiliations:** 1 Department of Radiology, University of Wisconsin, School of Medicine and Public Health, Madison, WI, USA; 2 Department of Medical Physics, University of Wisconsin, School of Medicine and Public Health, Madison, WI, USA

## Background

Four-dimensional flow-sensitive (4D flow) MRI opens the possibility for finding new, non-invasive measures of cardiac function, including KE. Studies using 4D flow MRI have reported correlations between disease states and altered KE profiles. However, the majority of these studies have relied on time-averaged (TA) segmentation rather than time-resolved (TR) segmentation largely because segmenting throughout the cardiac cycle (TR) is time-consuming and labor intensive. The purpose of this study was to determine if there are significant differences between KE profiles calculated using TR and TA segmentation of the right and left ventricles (RV, LV).

## Methods

Time-resolved 4D flow MRI (PC VIPR, phase contrast with vastly undersampled isotropic projection reconstruction) data were acquired from 10 healthy volunteers on a 3T scanner (MR750, GE Healthcare, Waukesha, WI). RV and LV volumes were segmented from TR and TA images using Mimics (Materialise, Leuven, Belgium). Velocity profiles were obtained using Ensight (CEI, Apex, NC), and the total KE in each ventricle was calculated from both the TR and TA segmentations using MatLab (The Mathworks, Natick, MA). KE profiles were then compared using paired Student's t-tests.

## Results

Small yet significant differences in calculated KE were observed between TR and TA segmentation. The peak systolic KE of the RV were 4.89±1.49 mJ using TR and 5.53±1.62 mJ using TA segmentation (P =0.016), while the peak systolic LV KE were 3.29±0.96 mJ and 4.16±1.26 mJ (P = 0.005). The peak diastolic RV KE were 3.33±0.90 mJ (TR) and 3.61±1.12 mJ (TA) (P = 0.082), while the peak diastolic LV KE were 4.90±1.49 mJ and 5.31±1.59 mJ (P = 0.044). The total integrated KE of the RV across the cardiac cycle were 1880±506 mJ (TR) and 2131±608 mJ (TA) (P = 0.004), while the integrated KE of the LV were 1775±510 mJ and 2080±658 mJ (P = 0.001).

## Conclusions

KE profiles for both the right and left ventricles were determined using TR and TA segmentation. Although qualitatively the two methods resulted in comparable results, TA segmentation consistently over-estimated KE, with differences between the two methods being more pronounced during systole and particularly in the LV. Therefore, for a qualitative analysis, using TA images for segmentation is recommended, while a more accurate, quantitative analysis requires a full TR segmentation.

## Funding

N/A.

**Figure 1:**
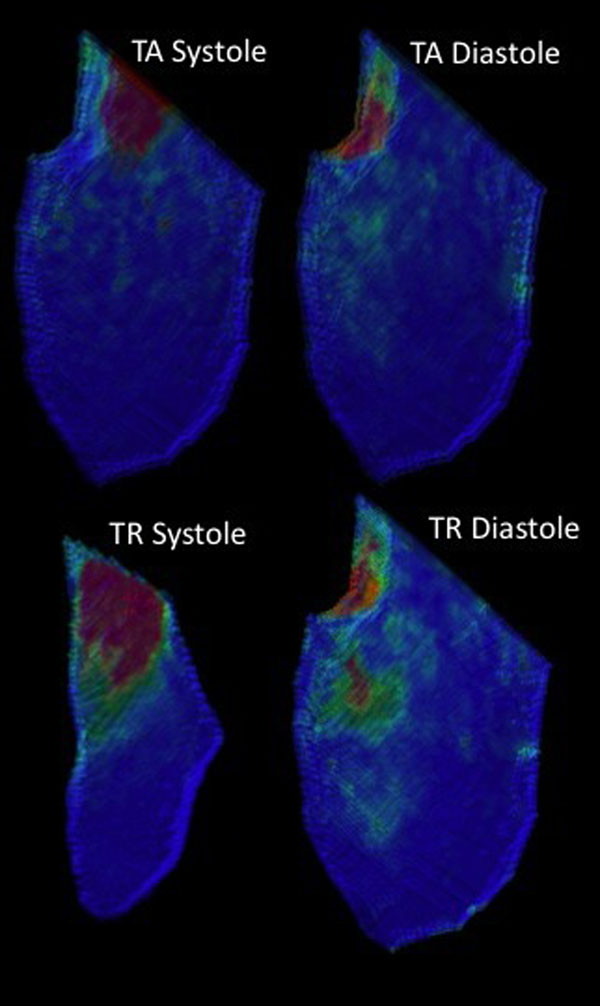
Visualization of volume and kinetic energy changes during diastole and systole for a particular subject, demonstrating the differences between time-average and time-resolve segmentation.

**Figure 2:**
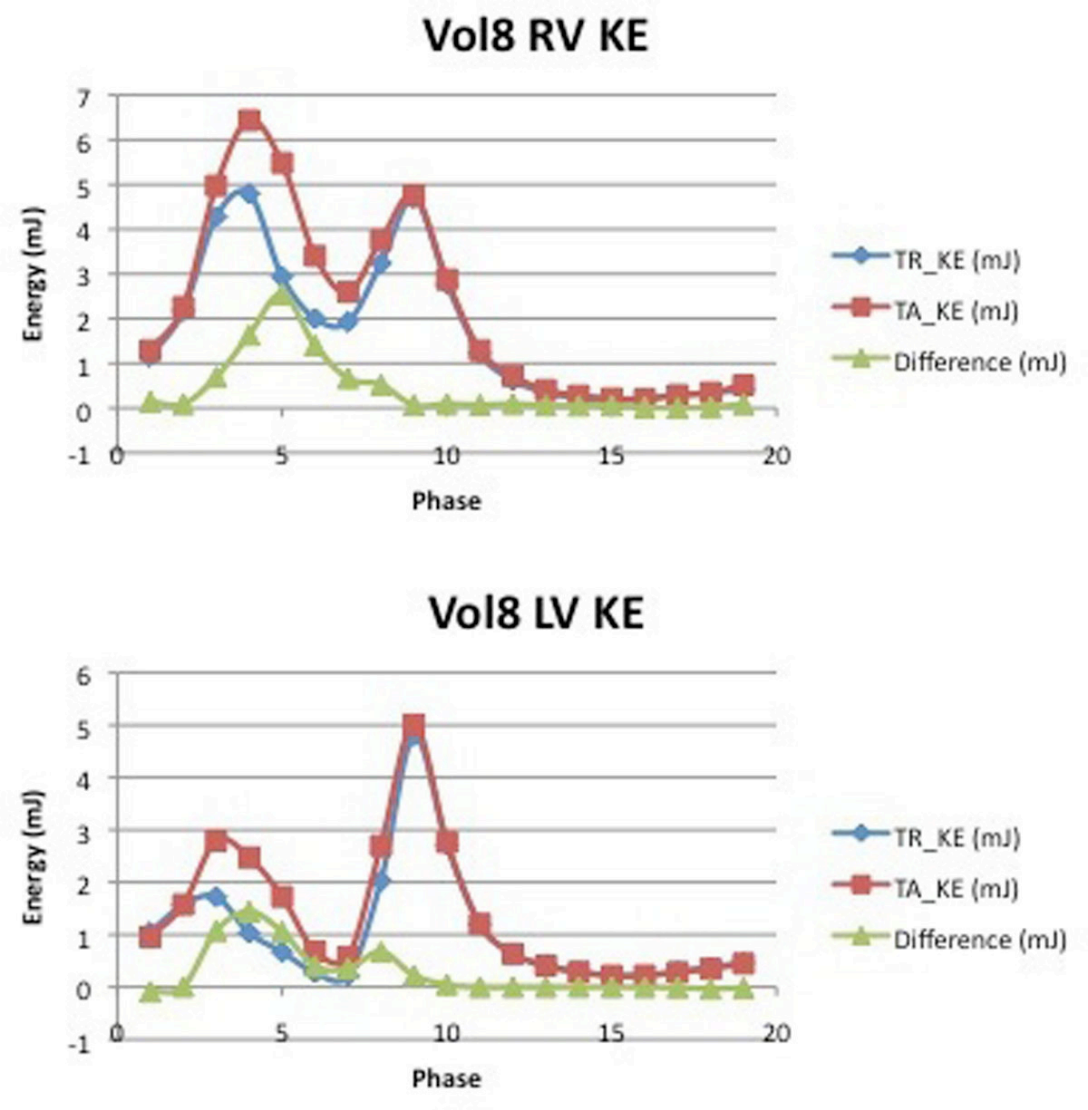
Plots of kinetic energy across the cardiac cycle for a particular subject (same subject as in Image 1), demonstrating the differences between LV and RV KE patterns, and between TA and TR segmentation results.

